# Reduced CYP2D6 function is associated with gefitinib-induced rash in patients with non-small cell lung cancer

**DOI:** 10.1186/1471-2407-12-568

**Published:** 2012-12-04

**Authors:** Tomohiro Suzumura, Tatsuo Kimura, Shinzoh Kudoh, Kanako Umekawa, Misato Nagata, Kuniomi Matsuura, Hidenori Tanaka, Shigeki Mitsuoka, Naruo Yoshimura, Yukimi Kira, Toshiyuki Nakai, Kazuto Hirata

**Affiliations:** 1 Department of Respiratory Medicine, Graduate School of Medicine, Osaka City University, Osaka, Japan; 2 Department of Central Laboratory, Graduate School of Medicine, Osaka City University, Osaka, Japan

**Keywords:** CYP2D6, Non-small cell lung cancer, Gefitinib, Erlotinib, Adverse events

## Abstract

**Background:**

Rash, liver dysfunction, and diarrhea are known major adverse events associated with erlotinib and gefitinib. However, clinical trials with gefitinib have reported different proportions of adverse events compared to trials with erlotinib. In an *in vitro* study, cytochrome P450 (CYP) 2D6 was shown to be involved in the metabolism of gefitinib but not erlotinib. It has been hypothesized that CYP2D6 phenotypes may be implicated in different adverse events associated with gefitinib and erlotinib therapies.

**Methods:**

The frequency of each adverse event was evaluated during the period in which the patients received gefitinib or erlotinib therapy. CYP2D6 phenotypes were determined by analysis of *CYP2D6* genotypes using real-time polymerase chain reaction techniques, which can detect single-nucleotide polymorphisms. The CYP2D6 phenotypes were categorized into 2 groups according to functional or reduced metabolic levels. In addition, we evaluated the odds ratio (OR) of the adverse events associated with each factor, including CYP2D6 activities and treatment types.

**Results:**

A total of 232 patients received gefitinib therapy, and 86 received erlotinib therapy. Reduced function of CYP2D6 was associated with an increased risk of rash of grade 2 or more (OR, 0.44; 95% confidence interval [CI], 0.21–0.94; *p = 0.03), but not diarrhea ≥ grade 2 (OR, 0.49; 95% CI, 0.17–1.51; *p = 0.20) or liver dysfunction ≥ grade 2 (OR, 1.08; 95% CI, 0.52–2.34; *p = 0.84) in the gefitinib cohort. No associations were observed between any adverse events in the erlotinib cohort and CYP2D6 phenotypes (rash: OR, 1.77; 95% CI, 0.54–6.41; *p = 0.35/diarrhea: OR, 1.08; 95% CI, 0.21–7.43; *p = 0.93/liver dysfunction: OR, 0.93; 95% CI, 0.20–5.07; *p = 0.93).

**Conclusions:**

The frequency of rash was significantly higher in patients with reduced CYP2D6 activity who treated with gefitinib compared to patients with functional CYP2D6. CYP2D6 phenotypes are a risk factor for the development of rash in response to gefitinib therapy.

## Background

Compared to cytotoxic agents, gefitinib and erlotinib are orally available epidermal growth factor receptor (EGFR) tyrosine kinase inhibitors (TKI) that prolong survival, have few hematological adverse events, and improve the quality of life in non-small cell lung cancer (NSCLC) patients with *EGFR*-active gene mutations [1-6]. The major adverse events that occur with gefitinib and erlotinib therapy are rash, liver dysfunction and diarrhea [1-5,7-10]. We are always faced with a decision to select between these drugs in clinical practice for patients with *EGFR*-active mutations. In general, erlotinib is associated with higher toxicity and lower tolerability than gefitinib because the dose of erlotinib used is nearly equal to the maximum tolerated dose, whereas the dose of gefitinib used is close to the minimum active dose [11,12].

Recent *in vitro* studies have reported different metabolic profiles of gefitinib and erlotinib for human cytochrome P450 (CYP) enzymes [13-15]. CYP3A4, CYP3A5, and CYP1A1 metabolize both erlotinib and gefitinib. However, CYP2D6 is involved in the metabolism of gefitinib but not erlotinib. It has been hypothesized that gefitinib therapy results in different adverse events compared to erlotinib therapy due to the CYP2D6 phenotype. To test this, we evaluated the adverse events of treatment with gefitinib and erlotinib. CYP2D6 phenotypes were determined from the *CYP2D6* genotypes using real-time polymerase chain reaction (PCR) techniques, which are able to detect single-nucleotide polymorphisms (SNPs).

## Methods

### Study subjects and data collection

Patients with advanced NSCLC who were treated with either gefitinib or erlotinib were retrospectively identified by analysis of patient information for subjects prospectively enrolled in the Medical Information System within Osaka City University Hospital between January 1999 and February 2012. This study protocol was approved by the ethics committee of Osaka City University (approval number, 1700). In our study, all patients received a single agent EGFR-TKI therapy. The frequency of each adverse event was evaluated during the period in which the patients received EGFR-TKI therapy. All living participants provided written informed consent. Formalin-fixed and paraffin-embedded tissues or blood samples (when tissues were not available) were collected. If the patients were dead, formalin-fixed and paraffin-embedded tissues were collected with the permission of the ethics committee. Adverse events were assessed according to the National Cancer Institute Common Terminology Criteria for Adverse Events (version 3.0). We defined liver dysfunction as one or more events of increased levels of aspartate aminotransferase (AST), alanine aminotransferase (ALT), or blood bilirubin. The frequency and severity of 3 major non-hematological toxicities, including rash, diarrhea, and liver dysfunction, were evaluated.

### Genotyping methods

Genomic DNA was extracted from peripheral blood or formalin-fixed and paraffin-embedded (FFPE) tissues using a QIAGEN QIAamp® DNA Blood Mini Kit (QIAGEN K.K., Tokyo, Japan) and a QIAGEN QIAamp® DNA FFPE Tissue Kit (QIAGEN K.K.), according to the manufacturer’s instructions. Extracted DNA samples were stored at −80°C before use. The DNA concentration was determined by measuring the optical density at 260 nm (Nano Drop® ND-1000, Thermo Fisher Scientific, Inc., Wilmington, DE, USA). In order to determine the *CYP2D6* polymorphisms, 4 SNPs of the *CYP2D6* gene, including rs1065852 (100C>T), rs5030865 (1758 G>A), rs16947 (2850C>T), and rs1135840 (4180 G>C), were measured by real-time PCR in order to evaluate the 5 mutated alleles: CYP2D6*1, CYP2D6*2, CYP2D6*10, CYP2D6*14A, and CYP2D6*14B. Genotyping was performed using Taqman® Drug Metabolism Genotyping Assays™ (Applied Biosystems Japan Ltd., Tokyo, Japan), according to the manufacturer’s instructions. The following reagents were used for amplification in a 10 μL reaction volume: 4.5 μL of DNA (around 50 ng), 0.5 μL of each *CYP2D6* primer and probe mixture (20×), and 5 μL of GTXexpress™ Master Mix. The thermal cycling conditions consisted of an initial 20 seconds at 95°C, followed by 40 cycles at 95°C for 15 seconds and at 60°C for 1 minute. Primers and probes were supplied by Applied Biosystems, Japan, Ltd as Drug Metabolism Genotyping Assays™. The assay IDs were C__11484460_40 for rs1065852, C_30634117D_30 for rs5030865, C__27102425_10 for rs16947, and C__27102414_10 for rs1135840. All assays were performed in 96-well plates. Plates were read on an Applied Biosystems 7500 Real-time PCR system using the Sequence Detection System Software (Applied Biosystems Japan Ltd.).

### CYP2D6 phenotype

The metabolic functions of CYP2D6 are generally categorized into 4 groups: ultra-rapid metabolizer (UM), extensive metabolizer (EM), intermediate metabolizer (IM), and poor metabolizer (PM) [16]. UM and EM result in normal or better function, and IM and PM result in reduced functions. *CYP2D6* alleles were assigned based on the determination of the appropriate key mutations. CYP2D6*1 and CYP2D6*2 have normal activities, CYP2D6*10 and CYP2D6*14B have impaired activities, and CYP2D6*5 and CYP2D6*14A have no activities [17-20]. Alleles containing additional copies of functional *CYP2D6* genes were categorized as UM. The EM included a combination of 1 or 2 functional alleles, such as CYP2D6*1 or CYP2D6*2, the IM phenotype included 2 impaired alleles, and the PM phenotype included two non-functional alleles. In this study, the CYP2D6 phenotype was categorized into 2 groups according to the metabolic levels: functional (UM and EM) or reduced groups (IM and PM). Unknown phenotypes with a combination of impaired and undetermined alleles, or 2 undetermined alleles, were excluded.

### Statistical analysis

Comparisons of the characteristics between patients treated with gefitinib or erlotinib were performed using Fisher’s exact tests. Hardy-Weinberg equilibrium was tested for with a goodness-of-fit χ^2^-test with 2 degree of freedom to compare the observed genotype frequencies among the subjects with the expected genotype frequencies. In order to identify the risk factors for the adverse events, gender, age, CYP2D6 activity, and stage were selected and estimated for their potential confounding effects on rash, diarrhea, and liver dysfunction by multivariate analysis. Unconditional logistic regressions were used to compute the odds ratios (ORs) and their 95% confidence intervals (CIs). All analyses were two-sided, and *p* values of less than 0.05 were considered statistically significant. The statistical analyses were performed with JMP 9 software (SAS Institute, Inc., Cary, NC, USA) and software R version 2.10.0 (The R Foundation for Statistical Computing, Vienna, Austria).

## Results

### Patient characteristics

**Figure 1:**
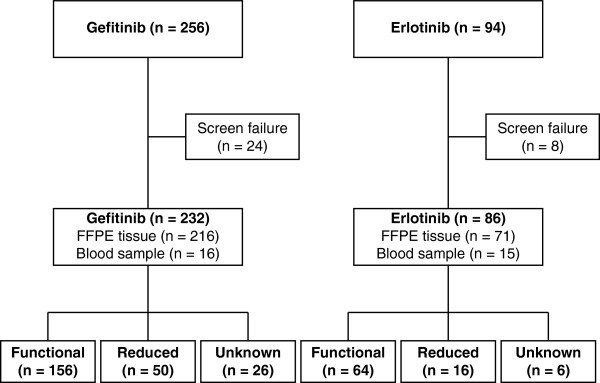
**Study profile.** Diagram shows patient disposition in the gefitinib and erlotinib treatment groups.

The study profile is illustrated in Figure 1. A total of 256 patients with advanced NSCLC who were treated with gefitinib, and a total of 94 patients with advanced NSCLC who were treated with erlotinib, were enrolled in the study. DNA samples were collected from 289 patients, including 232 patients who received gefitinib and 86 patients who received erlotinib. Among them, 29 patients who were treated with gefitinib were also treated with erlotinib at different times. DNA samples were not obtained from 24 patients who were treated with gefitinib and 8 patients who were treated with erlotinib because of screen failure. Genomic DNA was extracted from 232 samples, including 16 blood samples and 216 tissues, in the gefitinib group, and from 86 samples, including 15 blood samples and 71 tissues, in the erlotinib group.

The distributions of the patient characteristics among the study subjects are summarized in Table 1. Comparisons of the gefitinib and erlotinib groups that were representative of the cohort indicated that the gefitinib group had a higher rate of *EGFR* mutation-positive patients (gefitinib group, 95.6%; erlotinib group, 70.6%; *p < 0.001), and a higher rate of poor performance status (≥2) patients (gefitinib group, 25.4%; erlotinib group, 8.2%; *p = 0.001). There were no significant differences between the gefitinib and erlotinib groups in terms of age, sex ratio, histology, smoking status, stages, CYP2D6 functions, infection with the hepatitis B or C virus, or pretreatment liver function tests.

**Table 1:** Patient characteristics in the gefitinib and erlotinib groups

**Characteristics**	**Gefitinib (n = 232)**	**Erlotinib (n = 86)**	**p value**
**Median age (Range)**	67 (24–90)	66 (34–90)	0.36
< 70	139	57	
≥ 70	93	29	
**Gender**			0.90
Male	105	38	
Female	127	48	
**Histology**			0.32
Adenocarcinoma	223	80	
Squamous cell carcinoma	8	5	
Other	1	1	
**Smoking status**			0.38
Ever smoker	130	43	
Never smoker	102	43	
**ECOG performance status**			0.001
0	30	11	
1	143	68	
≥ 2	59	7	
**EGFR mutation status**			< 0.001
Positive	91	53	
Negative	4	22	
Unknown	137	11	
**Stage**			0.75
≤ IIIB	48	16	
IV	184	70	
**CYP2D6 activity**			0.42
functional	156	64	
reduced	50	16	
unknown	26	6	
**HBs antigen**			1.00
Positive	2	0	
Negative	211	85	
Unknown	2	1	
**HCV antibody**			0.10
Positive	15	5	
Negative	200	80	
Unknown	17	1	
**Pretreatment LFT**			0.42
normal	155	62	
abnormal	77	24	

*Fisher’s exact test were applied to compare patient characteristics.

*ECOG*, Eastern Cooperative Oncology Group; *EGFR*, Epidermal Growth Factor Receptor; *HCV*, hepatitis C virus; *HBs*, hepatitis B surface; *LFT*, liver function test; *CYP2D6*, cytochrome P450 2D6.

### Comparison of the adverse events of gefitinib and erlotinib

**Figure 2:**
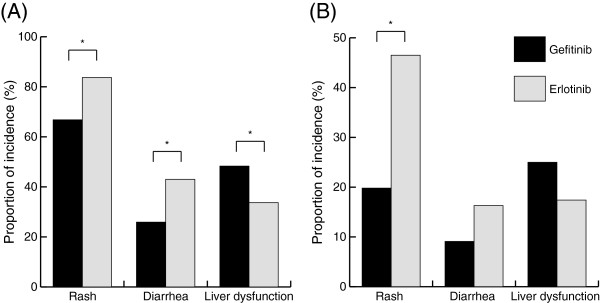
**Comparison of adverse events between the gefitinib and erlotinib groups.** The graphs show the proportion of adverse events in all grade (**A**), and in grade ≥ 2 (**B**). In all grade, the patients treated with gefitinib had a significantly higher frequency of liver dysfunction than did patients treated with erlotinib (*p = 0.003). In grade ≥ 2, liver dysfunction occurred significantly more often in the gefitinib group than in the erlotinib group (*p = 0.04).

Figure 2 shows the frequencies and severities of rash, diarrhea, and liver dysfunction. In the gefitinib treatment group, the rates of rash of all grades and of grade 2 or greater were 66.8% and 19.8%, those of diarrhea were 25.9% and 9.1%, and those of liver dysfunction were 48.3% and 25.0%, respectively. In the erlotinib treatment group, the rates of rash of all grades and of grade 2 or greater were 83.7% and 46.5%, those of diarrhea were 43.0% and 16.3%, and those of liver dysfunction were 33.7% and 17.4%, respectively. The patients treated with gefitinib had a significantly higher frequency of liver dysfunction than the patients treated with erlotinib (*p = 0.02). In contrast, the patients treated with erlotinib had a significantly higher frequency of rash and diarrhea than did the patients treated with gefitinib (*p = 0.003 and 0.04, respectively). Sixteen pneumonitis patients were observed only in the gefitinib group, and pneumonitis-related death was observed in 7 patients.

### *CYP2D6* alleles, genotype, and phenotype

The genomic DNA from a total of 289 patients was analyzed (Table 2). The distributions of *CYP2D6* alleles were as follows: CYP2D6*1, 236 alleles (40.8%); CYP2D6*2, 63 alleles (10.9%); CYP2D6*10, 211 alleles (36.5%); CYP2D6*14A, 1 allele (0.2%); and undetermined, 67 alleles (11.6%). In a total of 201 patients, genotyping predicted the normal function of CYP2D6*1/*1 in 67 patients, CYP2D6*1/*2 in 22 patients, CYP2D6*1/*10 in 72 patients, CYP2D6*1/*14A in 1 patient, CYP2D6*1/undetermined allele in 7 patients, CYP2D6*2/*2 in 9 patients, CYP2D6*2/*10 in 20 patients, and CYP2D6*2/undetermined allele in 3 patients. In a total of 58 patients, genotyping predicted reduced function associated with CYP2D6*10/*10. In a total of 30 patients, the genotypes was unknown with results of CYP2D6*10/ undermined allele in 3 patients, and 2 undermined alleles in 27 patients. The frequencies of CYP2D6*1, CYP2D6*2, and CYP2D6*10 were compared to the data previously reported in Japanese [21-23]. Compared to the Kubota’s, Nishida’s, and Tateishi’s reports, the genotype distributions of each phenotype among the patients were in Hardy-Weinberg equilibrium (*p = 0.69, 0.92, and 0.63, respectively).

**Table 2:** **Distribution of *CYP2D6 *alleles and genotypes**

**Alleles**	**Number**
CYP2D6*1	236 (40.8%)
CYP2D6*2	63 (10.9%)
CYP2D6*10	211 (36.5%)
CYP2D6*14A	1 (0.2%)
**Genotype**	**Number**
**Functional**	
CYP2D6*1/*1	67 (23.2%)
CYP2D6*1/*2	22 (7.6%)
CYP2D6*1/*10	72 (24.9%)
CYP2D6*1/*14A	1 (0.4%)
CYP2D6*1/undetermined	7 (2.4%)
CYP2D6*2/*2	9 (3.1%)
CYP2D6*2/*10	20 (6.9%)
CYP2D6*2/undetermined	3 (1.0%)
Total	201 (69.6%)
**Reduced**	
CYP2D6*10/*10	58 (20.1%)
**Unknown**	
CYP2D6*10/ undetermined	3 (1.0%)
undetermined/ undetermined	27 (9.3%)
Total	30 (10.3%)

*CYP2D6*, cytochrome P450 2D6.

### Comparison of adverse events among CYP2D6 phenotypes

**Figure 3:**
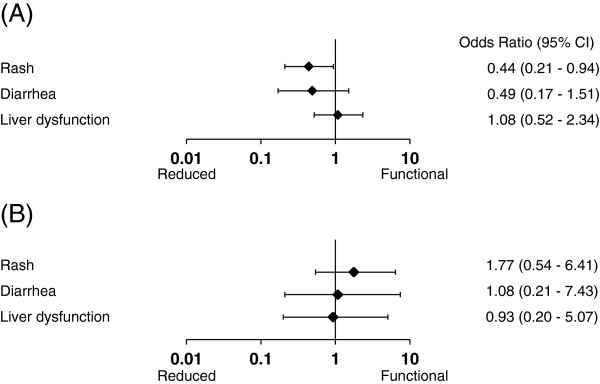
**The odds ratio of each adverse event in EGFR-TKIs.** The graphs show the forest plots for gefitinib (**A**), and for erlotinib (**B**). In the gefitinib cohort, reduced function was associated with an increased risk of rash (*p = 0.03). In the erlotinib cohort, there were no associations between any adverse events and CYP2D6 phenotypes.

Figure 3 shows forest plots of the odds ratio for risk factors determined by multiple logistic regression models. Each adverse event was divided into 2 groups: grade 0, 1 or grade ≥ 2. In the gefitinib cohort, the genotypes of 156 patients predicted normal function, and the genotypes of 50 patients predicted reduced function. Reduced function was associated with an increased risk of rash (rash: OR, 0.44; 95% CI, 0.21–094; *p = 0.03). Reduced function was not associated with an increased risk of diarrhea or liver dysfunction (diarrhea: OR, 0.49; 95% CI, 0.17–1.51; *p = 0.20, liver dysfunction: OR, 1.08, 95% CI, 0.52–2.34; *p = 0.84).

In the erlotinib cohort, the genotypes of 64 patients predicted normal function, and the genotypes of 16 patients predicted reduced function. There were no associations between any adverse events and CYP2D6 phenotypes (rash: OR, 1.77; 95% CI, 0.54–6.41; *p = 0.35, diarrhea: OR, 1.08; 95% CI, 0.21–7.43; *p = 0.93, liver dysfunction: OR, 0.93; 95% CI, 0.20–5.07; *p = 0.93).

## Discussion

We have demonstrated that reduced function of CYP2D6 in the gefitinib cohort was associated with an increased risk of rash of grade 2 or more. No associations were observed in the erlotinib cohort between any adverse events and CYP2D6 phenotypes.

Almost all patients received appropriate supportive care in their treatment. In our study, gefitinib treatment showed different characteristics to erlotinib treatment. In a previous report without patient selection, treatment with gefitinib was not associated with a significant improvement in survival compared to placebo therapy [4,5]. However, erlotinib has been shown to prolong survival in unselected and *EGFR* wild-type patients with NSCLC after first-line or second-line chemotherapy [6,24]. For these reasons, gefitinib is administered to *EGFR* mutation-positive patients and patients with a higher rate of poor performance status (≥2).

In our study, gefitinib treatment showed different adverse events to erlotinib treatment. Compared to the side effects of erlotinib, patients treated with gefitinib had a significantly higher frequency of liver dysfunction. In the gefitinib group, the rate of liver dysfunction of all grades in our study was 45.3%, including 19.0% of grade 1, 10.5% of grade 2, 14.7% of grade 3, and 1.1% of grade 4. In the erlotinib group, the rate of liver dysfunction in our study was 21.3%, including 8.2% of grade 1, 8.2% of grade 2, 4.9% of grade 3, and 0% of grade 4. With respect to gefitinib therapy, Maemondo *et al.* reported a rate of 55% of all grades of increased levels of aminotransferases, and the rate of grade 3 or 4 was 21.5% in a Japanese cohort [2]. Mitsudomi *et al.* reported a rate of 70.1% of all grades and a rate of 16.1% of grade 3 or 4 [25]. With respect to erlotinib therapy, an Asian phase III study showed a rate of 37% for all grades of increased levels of ALT, and a rate of 4% of grade 3 or 4 [26]. Our results were similar to those found in previous gefitinib and erlotinib phase III clinical trials in Asian subjects.

Liver dysfunctions induced by gefitinib were reported in a few cases in which hepatotoxicity caused by gefitinib declined when gefitinib was changed to erlotinib. First, Kijima *et al.* suggested the possibility that *CYP2D6* polymorphisms were related to gefitinib-induced hepatotoxicity. Their study described 3 cases with gefitinib-related hepatotoxicity whose genotypes were CYP2D6*1/*10, CYP2D6*10/*10, and CYP2D6*1/*5, with phenotypes of EM, IM and EM, respectively. Second, Takeda *et al.* reported a case and suggested that liver dysfunction was attributable to a gefitinib allergy on the basis of a positive drug lymphocyte stimulation test (DLST) [27]. In our study, the reduced function of CYP2D6 was not associated with an increased risk of liver dysfunction in the gefitinib cohort. Further analysis of the different metabolic profiles of CYP enzymes should be performed to clarify the metabolisms of gefitinib. The DLST of gefitinib may be considered of value in some patients with gefitinib-induced liver dysfunction.

The *in vitro* metabolism of gefitinib was investigated using human liver microsomes, and gefitinib metabolized mainly by expressed CYP3A4 produced a similar range of metabolites as liver microsomes [15]. When CYP3A4 function was low or inhibited by other drugs that inhibit CYP3A4, gefitinib metabolism that involves the formation of *O*-desmethyl-gefitinib and is determined by the CYP2D6 enzyme expressed in the liver was increased marginally [15]. Therefore, the CYP2D6 enzyme is important for the metabolism of gefitinib not only in patients with reduced CYP2D6 function, but also in patients with normal CYP2D6 function who take other drugs related to CYP3A4 inhibition. These patients treated with gefitinib may have severe skin rash due to decreased metabolism of gefitinib.

We evaluated 5 mutated alleles, CYP2D6*1, CYP2D6*2, CYP2D6*10, CYP2D6*14A and CYP2D6*14B, in 289 patients. The frequency of each allele was similar to those reported in previous Japanese studies. Two reports showed that the frequencies of the CYP2D6*1, CYP2D6*2, CYP2D6*5, CYP2D6*10, and CYP2D6*14 alleles were 40.1%, 12.9%, 6.2%, 38.6%, and 2.2%, respectively, in 162 Japanese [21], and 43%, 12.3%, 4.5%, 38.1%, and 0.7%, respectively, in 412 Japanese [22]. A further report showed that the frequencies of the CYP2D6*1, CYP2D6*2, CYP2D6*5, and CYP2D6*10 alleles were 42.3%, 9.2%, 6.1%, and 40.8%, respectively, in 98 healthy Japanese [23]. Taken together, the average frequencies of the functional and reduced function alleles in these studies were 54.2%, and 44.7%, respectively. Asians have a high frequency of the reduced function alleles that ranges from 43% to 47% [21-23,28]. However, in Caucasians, the functional and reduced function alleles represented a median frequency of 71% and 26%, respectively, in all cohort studies [16] and 68.8% and 25.36%, respectively, in German subjects [29]. Thus, Asians have a higher frequency of reduced function alleles than do Caucasians. In addition, population-related pharmacogenomics revealed a significant difference between Japanese and US patients in genomic distribution and genotype-related associations with patient outcomes for CYP3A4*1B and *ERCC2*[30]. These facts may explain the frequency differences of adverse events, particularly rash, that are based on race.

CYP2D6 metabolizes many clinically important drugs, including antidepressants, neuroleptics, beta-blockers, anti-arrhythmics, and anti-cancer agents. In breast cancer patients who were treated with tamoxifen, the CYP2D6 phenotype was associated with survival [31] and the concentration of the active tamoxifen metabolite, endoxifen [32]. Recently, in 2 single-agent studies with gefitinib in bronchioloalveolar and head and neck carcinomas, an association between the occurrence of skin toxicity and survival was found [33,34]. In our study, the subjects with reduced CYP2D6 function were associated with an increased risk of rash in the gefitinib cohort. Reduced CYP2D6 function may relate to longer overall survival as well as poor metabolism of gefitinib. A prospective large clinical trial is warranted to clarify these relationships.

Our findings had some limitations. The number of patients was too small to have sufficient power to detect significant differences in other adverse events between CYP2D6 phenotypes. This study was a retrospective analysis. The identification of adverse effects was prompted by monthly visits with hematological tests or a medical interview for onset of symptoms, although the hematological toxicities were in some cases diminished at the time of next visit because of self-judgment for discontinuation. The adverse events were generally controlled, except for interstitial lung disease. The relation between CYP2D6 and carcinogenic risk was not evaluated. The blood concentrations of gefitinib and erlotinib and the metabolites of gefitinib and erlotinib, were not measured. However, in clinical settings, it may be difficult to perform blood sampling at sufficient frequency to calculate area under the curve (AUC). We could not separate the UM cohort from the EM cohort. UM consists of CYP2D6*1 or CYP2D6*2, and this group was included with EM in this study. Other cytochrome P450 enzymes were not measured. The relationship between these concentrations and these enzyme phenotypes remain to be elucidated. Our study may provide useful information regarding drug selection for EGFR-TKIs. When gefitinib is administered in combination with drugs which inhibit CYP2D6 function, the frequency of severe rash by gefitinib may be increased. When the patients with unknown CYP2D6 phenotype, have a severe rash by gefitinib, the change of gefitinib to erlotinib may be sometimes useful, because the patient may have reduced CYP2D6 function and CYP2D6 is not affected the erlotinib metabolism. CYP2D6 genotyping and an understanding of CYP2D6 function may be helpful in predicting rash during gefitinib therapy.

## Conclusions

We conclude that patients with reduced CYP2D6 activity treated with gefitinib had a significantly higher frequency of rash than did patients with functional CYP2D6. CYP2D6 phenotypes are a risk factor for the development of rash in gefitinib therapy. In contrast, no associations were found between the toxicity of liver dysfunction and CYP2D6 activity in patients treated with gefitinib. In our knowledge, our study is the first report that CYP2D6 phenotypes are related to severity of rash by gefitinib. Further clinical studies that include prospective investigations in a large patient population with pharmacokinetics/pharmacodynamics analyses, and that include detailed information regarding CYP2D6 genotype, phenotype and activity, should be conducted.

## Competing interests

The authors have made no disclosures.

## Authors’ contributions

TS and TK conceived of the study, participated in the design of the study, acquisition of data, performed the statistical analysis, drafted and revising the manuscript. SK conceived of the study, participated in the design of the study, acquisition of data and revising the manuscript. KU, MN, MK, HT, SM, NY, NT were involved in acquisition of data. YK carried out real-time PCR methods. KH participated in the design of the study, acquisition of data and revising the manuscript. All authors have read and approved the final version of the manuscript.

## Pre-publication history

The pre-publication history for this paper can be accessed here:

http://www.biomedcentral.com/1471-2407/12/568/prepub
